# Assessment and Efficacy of Low-Dose CT Screening and Primary Care Providers Perspective on Lung Cancer Screening: An Institutional Review

**DOI:** 10.7759/cureus.13778

**Published:** 2021-03-09

**Authors:** Manan Shah, Phani K Surapaneni, Kirat Sandhu, Saba Shafi, Temidayo Abe, Sanjay Jain, Gabriela Oprea, Judith Volcy

**Affiliations:** 1 Internal Medicine, Morehouse School of Medicine, Atlanta, USA; 2 Pathology and Laboratory Medicine, Yale School of Medicine, New Haven, USA; 3 Hematology and Medical Oncology, Morehouse School of Medicine, Atlanta, USA; 4 Pathology and Laboratory Medicine, Emory University School of Medicine, Atlanta, USA

**Keywords:** cancer, screening, prevention

## Abstract

Lung cancer is the most common cause of death in both men and women. The United States Preventive Services Task Force (USPSTF) recommends annual lung screening with low-dose computed tomography (LDCT) chest for individuals aged 55-80 who have a 30 pack-year smoking history and currently smoke or have quit within the past 15 years. We reviewed the electronic medical records of patients visiting our outpatient clinic over a period of one year. We included all eligible individuals according to USPSTF guidelines for LDCT to identify screening rates at our institution. All primary care physicians, including residents and attendings, were given a prepared questionnaire to understand their beliefs and concerns with the implementation of this program. A total of 13,500 patients visited the outpatient clinic and 1178 were eligible for LDCT. Forty-five percent (45%) of patients received LDCT screening, which was higher than the national average of 2%-5%. A total of 50 primary care providers were included in the survey. The majority of the providers were aware of the USPSTF guidelines and believed that patients with multiple comorbidities and insurance issues were barriers in initiating LDCT screening. Lung cancer screening is an important component in cancer preventive strategies. Widespread awareness among the primary care providers and the public is extremely necessary for improving the use of LDCT.

## Introduction

Lung cancer is the leading cause of death in both men and women in 2020, with about 135,000 deaths in the United States [1]. Studies have shown that only 15% of lung cancers are detected at an operable stage, resulting in overall poor survival [2]. Smoking has been associated with a significant risk for developing lung cancer. Effective screening strategies, especially with low-dose computed tomography (LDCT), aims at early detection of lung cancer thereby decreasing the mortality rate.

The National Lung Screening Trial (NLST), a randomized controlled trial was among the initial studies that reported a 20% lung cancer-specific mortality and overall mortality reduction with annual LDCT [3]. Largely based on the results of this study, various societies, including the American Society of Clinical Oncology (ASCO) and the United States Preventive Services Task Force (USPSTF), made recommendations for the screening of lung cancer using LDCT. The USPSTF published guidelines recommending LDCT in individuals between the ages of 55 and 80 years, with at least a 30-pack year history of smoking who either continue or have quit within the last 15 years. In 2020, USPSTF made grade B recommendations modifying the age of LDCT to 50-80 years with at least a 20-pack year history of smoking [4].

In the United States, it was estimated that 8.07 million people were eligible for LDCT screening in the year 2018-2019 nationwide, with a screening rate of approximately 5%. The better-performing states were Massachusetts, Michigan, and Washington [5]. However, various barriers were noted for the very low screening rates and proper implementation of this program nationwide. These barriers include lack of patient awareness, resource allocation, insurance issues, and physician education. Among them, primary care provider awareness of this program was found to be an important factor for which accurate data is not available. Insurance issues and policies toward LDCT implementation is also thought to be an important cause for below-average utility nationwide. Hence, we prepared a questionnaire to assess the beliefs and barriers among primary care providers in our institution and assess the LDCT screening rates in our hospital, which is the largest tertiary care center in the region with an established pulmonary nodule clinic. The aim was to enhance the program in our institution by finding important barriers and compare our screening rates with the national average. We also aimed at giving recommendations that could be implemented nationally.

## Materials and methods

Participants

To assess the screening rates, based on the USPSTF guidelines before the 2020 updated recommendations, we identified eligible candidates visiting the outpatient clinic of our institution from January 2019 to January 2020. We took into consideration baseline characteristics like age and sex, history of smoking pack-year (number of packs of cigarettes smoked per day multiplied by the number of years the person smoked), and information regarding receiving yearly LDCT if eligible. The assessment of belief, knowledge, and barriers toward LDCT screening among primary care providers was done using preformed questionnaires that were methodically prepared. Primary care providers included physician attendings, residents, and nurse practitioners working in our outpatient clinic. The study was approved by the institutional review board.

Questionnaire

We included various sections in our questionnaire for better clarity on beliefs and barriers among participants.

Awareness of guidelines

Participants were asked about their current knowledge on the recommended guidelines set by USPSTF for LDCT to assess if they knew who and when to order it for their patients. Questions also included knowledge of LDCT among their patients. Participants were asked if they required additional information on LDCT to strongly prescribe it without any hesitation.

Barriers to LDCT program implementation

Nine potential barriers were included in addition to a comment section at the end to include other factors that the participants found potentially important. They were asked to mark all options that were applicable according to their views. The potential barriers included multiple medical conditions among the patients that affected the participation in the LDCT program, lack of staff to implement the program, lack of patient interest, lack of reimbursements, insurance/Medicare issues, inadequate hospital policy/facility, and legal issues.

Future interventions

Participants were asked about the possible interventions that could aid in the improvement of screening rates and better implementation of the LDCT program. These included creating a separate LDCT registry, advancing the electronic medical record system to automatically identify individuals during their outpatient visit according to the guidelines, continuing education programs on LDCT and insurance policies for updates, and creating a dedicated lung clinic in the institution.

Beliefs on screening programs

Participants were asked about their potential beliefs on screening programs and if LDCT screening needs more awareness among physicians and the public.

Statistical methods

Descriptive statistics were used to characterize the beliefs and barriers and to understand the potential causes of LDCT under utility. Baseline characteristics like age, sex, and smoking history were noted. Logistic regression was used to identify predictors if any. Weighted percentages were used to summarize data. All the analysis was done using SAS version 9.4 (SAS Institute Inc, Cary, North Carolina).

## Results

Institution LDCT screening rate (SR)

**Figure 1:**
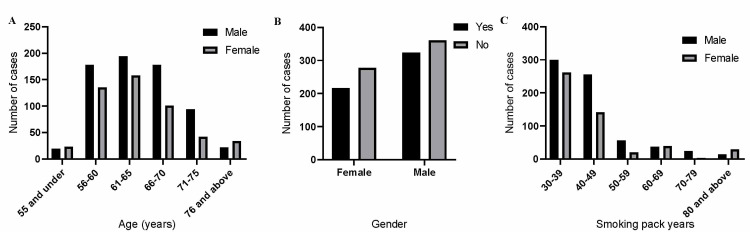
Demographic data of our patient population Bar diagrams showing (A) Age, (B) Gender, and (C) Smoking pack years in our study population. This also includes information for both males and females.

A total of 13,500 patients visited the outpatient clinic at our institution between January 2019 and January 2020, out of which 1178 were eligible for LDCT as per USPSTF and ASCO guidelines. The majority of the eligible candidates 685/1178 (58%) were males. The mean age was 63 years for both males and females. The majority of our patients, 261/493 (52%) of females, had a smoking pack-year history between 30 and 39 while 299/685 (43%) males had similar smoking pack-years. Active smokers constituted 60% of our patient population. The calculated LDCT screening rate, that is, patients that received LDCT/total eligible candidates x 100 was 540/1178 (45%). Females had an LDCT screening rate of 43% (216/493) while males had a screening rate of 47% (324/685). Our LCDT screening rate was higher than the national average of 2%-5%. Our institution has an established pulmonary nodule clinic. The inappropriate testing rate was only 3% (Figure 1).

Questionnaire results

**Figure 2:**
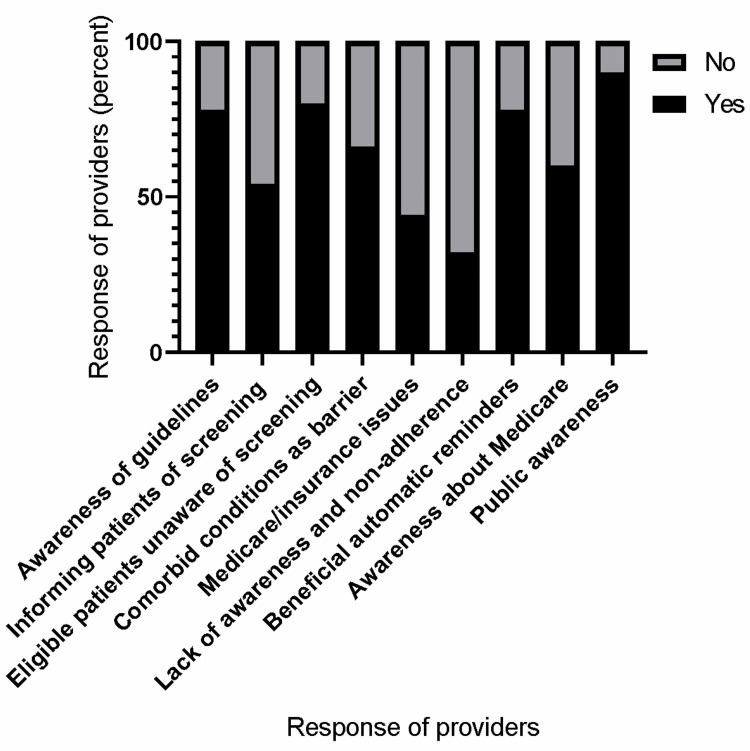
Response of providers Primary care providers' (N-50) perceived beliefs and barriers toward LDCT cancer screening

A total of 50 primary care providers, including physician attendings, internal medicine residents, and nurse practitioners were included in the study. A well-drafted questionnaire was prepared with the help of various National Cancer Institute (NCI) cancer-based screening practices questionnaires (Figure 2).

A) Consensus on Awareness of Guidelines

Most primary care providers, approximately 78%, reported that they were aware of the USPSTF/ASCO guidelines for LDCT screening. Most of the providers clearly mentioned two out of three components of LDCT screening guidelines, which included age group, smoking pack-years, and smoking status (active vs smoking history within 15 years). Fifty-four percent (54%) of providers reported that they informed their eligible patients of LDCT screening regularly. Eighty percent (80%) of the providers reported that eligible patients were not aware of the LDCT screening program when informed.

B) Consensus on Barriers to LDCT Program Implementation

Our providers reported that multiple co-morbid medical conditions were an important barrier to prescribing LDCT, with nearly 66% reporting it. This was followed by Medicare/insurance issues, which constituted 44%. Lack of public awareness and non-adherence to follow-up appointments were also thought to be important barriers to the successful implementation of this program, with nearly 32% of the providers endorsing this.

C) Consensus on Future Interventions

Approximately 78% of the providers believed that creating automatic and timely reminders based on guideline components on the electronic medical record system (EMR) will be beneficial for improving future screening rates and avoiding missing LDCT screening for eligible candidates. Sixty percent (60%) reported that creating awareness through educational programs among providers, especially with respect to Medicare policies and guideline updates, will go a long way in improving national screening rates apart from our institutional screening rate. Having dedicated lung nodule clinics in almost all institutes nationwide was also advocated by the majority of our providers.

D) Beliefs on Screening Programs

Nearly 90% of our providers believed that screening programs play an important role in cancer prevention. Unlike cervical cancer prevention with PAP smears and mammograms for breast cancer, which has considerable public awareness, LDCT for lung cancer is still in its primitive stage. Lack of public awareness was advocated to be an important factor for low screening rates nationwide in comparison to other cancer screening programs. Nationwide public meetings and advertisements on social media and public television are a few acceptable interventions.

## Discussion

The aim of our study was to assess the LDCT screening rates at our institution, which has a well-established lung nodule clinic, and to compare it with national averages. Our aim was to assess if our current model with an established clinic and necessary support staff would be beneficial for improving national averages. The LDCT screening rate (SR) at our institution was 45%, which was higher than the national average of 2%-5%. The inappropriate testing rate was only 3%. We also found that yearly LDCT follow-up studies were appropriate, for which data are currently being compiled at our institution. We strongly believe and recommend our model of establishing lung nodule clinics with appropriate support staff, including radiologists, pulmonologists, nurse practitioners, and research assistants in all states and major centers, which will benefit our national averages and bring the LDCT screening program on par with other cancer programs. The LDCT screening program has been underestimated even when various trials have shown a 20% mortality benefit [3,5].

Our study also aimed at understanding the beliefs and concerns of our primary care providers, as they form the first contact for most of our patients and are responsible for ordering LDCT for lung cancer screening. While most of our providers were aware of LDCT guidelines, the major concerns for our providers were Medicare and insurance issues. Few patients were personally willing to pay to get LDCT, the majority of them were dependent on insurance clearance [6]. Our study was conducted just before new USPSTF Grade B recommendations in 2020. We strongly propose that private insurance companies and centers for Medicare and Medicaid relax their stringent rules on insurance coverage for LDCT, which also include not deciding coverage based on co-morbid conditions and presuming patient compliance, which has been frequently the reasons for denying coverage. These changes should provide more autonomy to providers in prescribing LDCT.

Most providers in our survey believed that the LDCT screening program was publicized at a lower rate in comparison to breast screening and PAP screening among the public and physicians thereby contributing to its lower screening rates. Providers also believed that it might be less effective. However, data available to compare it with other screening programs in terms of the number needed to screen (NNS) to prevent one death is comparable, and this data needs to be circulated for better understanding among the community. The NNS for LDCT is 320 based on three annual scans, which compares favorably with mammograms, with an NNS of 1339 over a period of 11-20 years and NNS of 274 for hypertension to prevent cardiovascular death, which was lower than that of LDCT [7].

Our study and providers believe and recommend using technology to identify and remind physicians about LDCT screening in patients who meet the guidelines, especially during their outpatient visits. This could be done by updating the electronic medical record system (EMR) with alerts based on patients' smoking history and age-based reminders about necessary and due screening tests. Updating eligible patients with access to my-chart and email about the LCDT screening program and its benefits can help us create awareness. Creating awareness through media and technology can play an important role in improving our national average in a methodical manner.

To our knowledge, our study is among the few studies that calculated the annual LDCT screening rates at an institutional level. Our institution, being a tertiary care center in a metropolitan area, with an established lung nodule program and data supporting it, could act as a model for national centers and policy forming bodies. We would repeatedly reiterate the importance of this program in decreasing cancer mortality. Consistent with previous studies, we took into consideration our primary care providers to report their attitudes, which could be one of the important factors limiting the use of the LDCT program at multiple levels [8]. Creating a conscious plan with residents and young physicians, especially in the field of internal medicine, to promote and remove any bias against LDCT is extremely necessary and could only be possible by having a structured lung program at almost every institution. Further studies involving centers with established lung nodule clinics and programs to arrive at a broader consensus are essential. Involving providers from all these centers will give us newer ideas that could be targeted at a national level for better coverage of LDCT in lung cancer prevention [9-13].

In conclusion, we advocate involving patients and studying their beliefs and understanding toward LDCT as well, which we intend to conduct in the future. Awareness of the LDCT screening program and bringing it at par with other cancer and non-cancer screening programs remains our primary goal. We also advocate strengthening preventive oncology at a national level, as this forms a strong component in our fight against cancer [14-15].

## Conclusions

Lung cancer screening is an important component of cancer prevention strategies. However, screening rates are very low in comparison to other cancer screening programs. Various barriers have been noted in implementing this program. Widespread awareness among the primary care providers and the public is extremely necessary for improving the use of LDCT.
